# The Genus *Parabacteroides* Is a Potential Contributor to the Beneficial Effects of Truncal Vagotomy–Related Bariatric Surgery

**DOI:** 10.1007/s11695-022-06017-9

**Published:** 2022-05-11

**Authors:** Dong Liang, Xin Zhang, Zhaorui Liu, Rui Zheng, Longjiang Zhang, Dong Yu, Xiaojun Shen

**Affiliations:** 1 Translational Medicine Research Center, Naval Medical University, Shanghai, 200433 People’s Republic of China; 2 Department of General Surgery, Chang Hai Hospital, Naval Medical University, Shanghai, 200433 People’s Republic of China

**Keywords:** Gut microbiota, Sleeve gastrectomy, Truncal vagotomy, *Parabacteroides*

## Abstract

**Purpose:**

Evidences about the gut microbiota role in weight loss after bariatric surgery (BS) are growing. The objective of this study was to observe the changes of gut microbiota after sleeve gastrectomy (SG) and SG plus truncal vagotomy (SG-TV) and identify specific microbes that may contribute to the improvement of obesity after surgeries.

**Materials and Methods:**

Forty high-fat diet-induced obesity (DIO) mice were randomized to SG, SG-TV, or sham operation (SH) groups. Body weight (BW) and fast blood glucose (FBG) were measured before and 1, 2, 4, 8, and 12 weeks post-operatively. Fecal samples were collected before and at post-operative week 12 and profiled using 16S rRNA relative and absolute quantitative sequencing.

**Results:**

After the surgery, the SG and SG-TV surgeries significantly reduce BW and FBG levels compared with SH, and the SG-TV achieved better effects than SG. A decreasing trend in alpha diversity of gut microbiota and significant changes in taxonomic composition were observed after surgeries. Then, we identified a set of microbes and pathways significantly different in abundance after BS. The genus *Parabacteroides* and one pathway (polyketide sugar unit biosynthesis) increased in SG-TV group specially, which was also negatively correlated with BW and FBG.

**Conclusion:**

SG and SG-TV indeed achieve effects of weight loss, but TV could enhance the efficacy of SG. The identified different microbes and pathways, like *Parabacteroides*, polyketide sugar unit biosynthesis, may partly mediate the beneficial effects of BS, and thus possibly contribute to the development of novel bacteria-based therapeutic approaches.

**Graphical Abstract:**

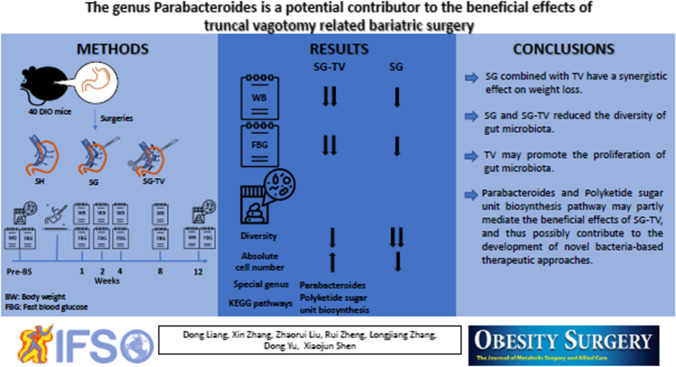

**Supplementary Information:**

The online version contains supplementary material available at 10.1007/s11695-022-06017-9.

## Introduction

The incidence of obesity is increasing at an unprecedented rate, and morbid obesity is currently a global health problem [1]. Bariatric surgery (BS) is currently the most effective treatment for obesity, and the application of sleeve gastrectomy (SG) exceeds 50% [2]. Besides, because of the role of vagus nerve in the regulation of appetite and obesity, it is increasingly recognized in the treatment of obesity [3].

Regardless of the method of BS, the gastrointestinal system will be affected, followed by changes of microbial community in the stomach and gut [4]. Several studies have evaluated the relations between gut microbiota changes after BS [5–8]. Animal and anthropological studies have reported the gut microbial changes after SG [9–15]. For example, Lu et al. found that the SG procedures lead to the increase of phylum *Verrucomicrobia* compared with sham group [10]. Sanchez-Alcoholado et al. found that the patients with severe obesity subjected in SG showed higher levels of *Akkermansia, Eubacterium*, *Haemophilus*, and *Blautia* [4]. However, controversial results were achieved from different literatures. Take phylum *Proteobacteria* for instance, some studies have shown a decrease in the abundance of *Proteobacteria* after SG, while some other studies concluded SG leads to an increase or no changes in *Proteobacteria* [5, 8, 13]. Given the controversial results of these researches, the present study will investigate the effects of SG on gut microbiota in DIO mice.

The vagus nerve is a central factor in the microbiome-gut-brain axis, microbes could communicate to the brain through the vagus nerve [16–18]. Evidences have showed that the vagus nerve could regulate eating behavior and BW [3, 19–21]. Actually, truncal vagotomy (TV) was promoted as a weight-loss procedure and resulted in good effects as early as 40 years ago [22, 23], although lost focus with the development of SG and Roux-en-Y gastric bypass (RYGB) subsequently. In recent years, with increasing attention of vagus nerve on weight regulating, several studies have reported that TV or SG-TV resulted in drastic reductions of BW [24, 25]. However, there is no study reported the effect of TV and TV-related BS on changing of gut microbiota. It remains to elucidate whether the vagus nerve and its related microbes could affect the effects of BS.

In this study, 40 high-fat diet-induced obesity (DIO) mice were used to perform different BSs, the body weight (BW) and fast blood glucose (FBG) were measured before and 1, 2, 4, 8, and 12 weeks post-operatively. The gut microbiota was also profiled before and after surgeries. We found SG and SG-TV indeed result in distinct changes in gut microbiota composition and metabolic pathways. And a specific genus *Parabacteroides* and one pathway (polyketide sugar unit biosynthesis), were identified to increase in the SG-TV groups, suggesting an important role in weight loss.

## Materials and Methods

### Animals

Ten-week-old male specific pathogen-free (SPF) mice (Jackson Laboratory, USA) were individually housed under a stable room temperature and relative humidity in a 12/12 h light/dark cycle. All the animals had free access to tap water and food unless otherwise stated. The mice were fed with a high-fat diet (Research Diets D12492, 60 kcal%) for 8 weeks to make them acquire diet-induced obesity (DIO). Subsequently, the DIO mice were divided to three groups randomly: SH (*n* = 11), SG (*n* = 19), and SG-TV (*n* = 10).

### Surgical Interventions

Before the surgical operation, the mice were fasted 14 h with water available freely, and the BW and FBG levels were measured at baseline before surgery and on a weekly basis after surgery. Mice were anesthetized and then subjected to SH, SG, and SG-TV surgeries according to the group distribution. For SG, the procedure included a midline incision was made 1 cm below the xiphoid; the greater curvature of the stomach (approximately 80% of the gastric volume) was removed; the remnant stomach was closed using 7-0 silk sutures in a continuous manner. For SG-TV, in addition to the above operations, we made an incision in gastrohepatic ligament to expose the esophagus and the trunk of the vagus nerve, and then cutted off both the dorsal and ventral trunks above the point of bifurcation into the celiac and gastric or hepatic and accessory celiac branches, respectively. For sham operation (SH), the mice underwent laparotomy to expose the stomach, esophagus, and vagus trunk around the esophagus. No other procedures were carried out, but the exposure time and the incision were the same as SG and SG-TV groups.

## Fecal Sampling, Processing, and Analysis

Fecal pellets were collected before and 12 weeks post-operatively. All the fecal samples were collected with sterile EP tubes and immediately stored at −80 °C prior to processing. The DNA of total bacteria in fecal samples were extracted with QIAamp Fast Stool DNA kit (Qiagen, Germany). The DNA samples were sent to Genesky Biological Technology Co., Ltd. (Shanghai, China) for 16S rRNA relative and absolute quantitative sequencing. The general description of the 16S rRNA absolute quantification sequencing was outlined in previous studies [26, 27]. Briefly, the V3-V4 region of bacteria 16S ribosomal RNA gene were chosen for amplification and sequenced on Illumina MiSeq. Nine different spike-in sequences with at least four different concentrations (10^3^, 10^4^, 10^5^, and 10^6^ of copies of internal standards) were added to the sample DNA pools. Spike-in sequences contained conserved regions identical to those of selected natural 16S rRNA genes and artificial variable regions different from nucleotide sequences in the public databases, which worked like internal standard and allowed the absolute quantification across samples.

## Statistical Analysis

The data are expressed as the mean ± standard deviation (SD). BW and FBG levels were evaluated by using one-way analysis of variance (ANOVA), and statistical analyses were performed using IBM SPSS 19.0 version software. The Alpha diversity analysis and principal component analysis (PCA) were performed by R (version 4.0.5). The differential abundance of taxa between groups was identified through linear discriminant analysis (LDA) effect size (LEfSe) algorithm. Only taxon with LDA score > 2 or *p* < 0.05 (Kruskal–Wallis test) was considered significantly enriched.

## Results

### Different Surgeries Reduce BW and FBG

**Fig. 1:**
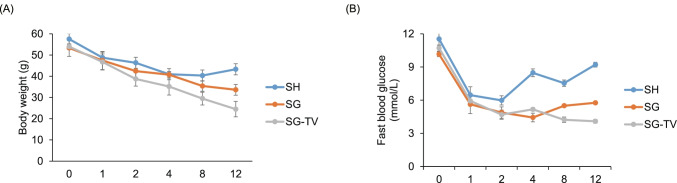
Effects of SH, SG, and SG-TV on body weight (A) and blood glucose (B). Data are expressed as mean ± SEM. Statistical differences are analyzed by one-way analysis of variance (ANOVA) with LSD post hoc test

BW and FBG of mice were measured before and 1, 2, 4, 8, and 12 weeks post-operatively. We found the BW of mice steadily decreased, and the FBG level dropped dramatically in the first 2 weeks, then slowed down or rebound in 3 groups (Fig. 1). At week 12 post-operatively, the mean BW of mice in the SG-TV group was lowest (24.50 ± 2.52 g), followed by SG (33.62 ± 3.63 g), and SH group was the highest (43.29 ± 2.35 g). Moreover, a same trend was observed in the FBG level. Notably, the FBG level of mice in the SH group had a trend to recover to the pre-operative level.

Overall, the SG and SG-TV surgeries could achieve the effect of weight loss significantly, but the effect of SG-TV was better than that of SG, suggesting the synergistic effect of SG combined with TV in weight loss.

## Different Surgeries Reduce the Diversity of Gut Microbiota

**Fig. 2:**
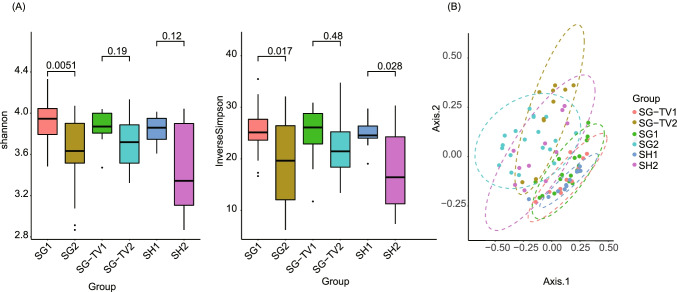
(A) Alpha diversity alteration after different bariatric surgeries. (B) Principal component analysis (PCA) on 40 fecal samples of each group. Each dot represents a sample, and different colors denote the samples collected from the different groups. 1, pre-surgery; 2, post-surgery

Diversity analysis showed a trend of decrease in bacterial diversity after surgeries. Simpson and Simpson indexes were significantly decreased after SG (*p* < 0.05) (Fig. 2A). PCA analysis indicated that the gut microbiota in the pre-surgery groups were distinctly dissimilar to that in the post-surgery groups (Fig. 2B), which indicated a great change of gut microbiota compositions caused by surgeries.

## Different Surgeries Altered the Taxonomic Composition of Gut Microbiota

**Fig. 3:**
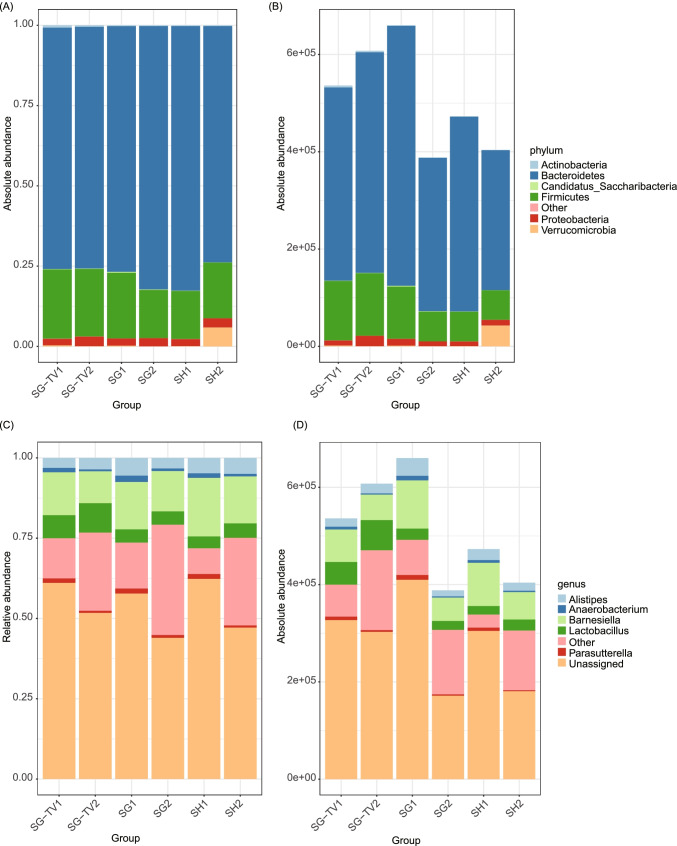
The absolute and relative abundances of the major bacteria at phylum and genus levels. 1, pre-surgery; 2, post-surgery

The composition of gut microbiota was analyzed from two perspectives: the relative abundances and absolute abundances of microbiota. The dominant phyla of gut microbiota were *Bacteroidetes*, *Firmicutes*, *Proteobacteria*, *Actinobacteria*, *Candidatus Saccharibacteria* and *Verrucomicrobia* (Fig. 3A). Absolute abundance analysis indicated that the absolute cell number obviously decreased in SH (0.85-fold) and SG (0.58-fold) groups, whereas increased in SG-TV group (1.13-fold), suggesting that TV may promote the proliferation of gut microbiota. But focused on specific phylum, we found that the total cell number of *Proteobacteria* and *Verrucomicrobia* increased apparently after SH. Similar results were observed at genus level (Fig. 3B), and the genera *Anaerobacterium*, *Barnesiella* and *Parasutterella* were found to decrease in SG-TV group after surgery. While for the relative abundance, some opposite results were observed. The relative abundance of phyla *Bacteroidetes* and *Proteobacteria* increased in SG group. Abundances of genus *Alistipes* increased in the SH group, while *Anaerobacterium* decreased in SG.

Considering that the absolute abundance could uncover the comprehensive dynamics of different bacterial communities, the subsequent analysis in this study was based on absolute abundance data [28].

## The Microbiota Associated with BW and FBG

**Fig. 4:**
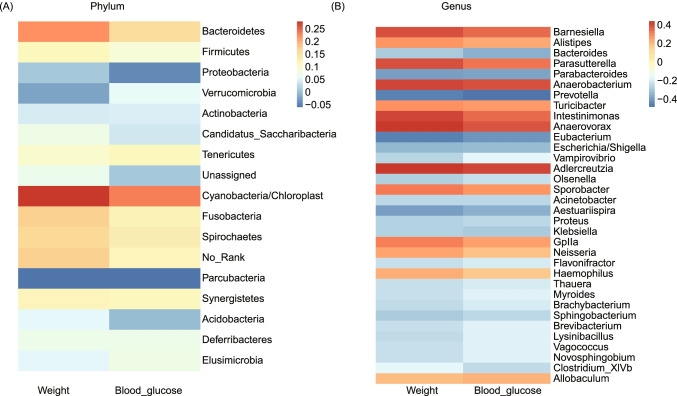
Correlation between post-operative weight or blood glucose and gut microbiota at phylum (A) and genus (B) levels. Only the genus with significant differences were showed (*p* < 0.05)

We further explored whether the change of gut microbiome have effects on weight loss and FBG decrease, and Pearson correlation analysis was conducted based on the data of 12 weeks post-operatively (Fig. 4). Weak correlations between BW or FBG and the abundance of gut microbiota were identified. Moreover, it is clear to see that the same correlation trend occurred in weight and FBG. At phylum level, *Cyanobacteria/Chloroplast* was the only phylum with significant correlation (weight *r* = 0.282, *p* = 0.011; FBG *r* = 0.233, *p* = 0.037) (Fig. 4A). At genus level, *Eubacterium*, *Prevotella* and *Parabacteroides* were the most negatively correlated with weight and FBG (Fig. 4B).

## Enriched Microbes in Different BSs

**Fig. 5:**
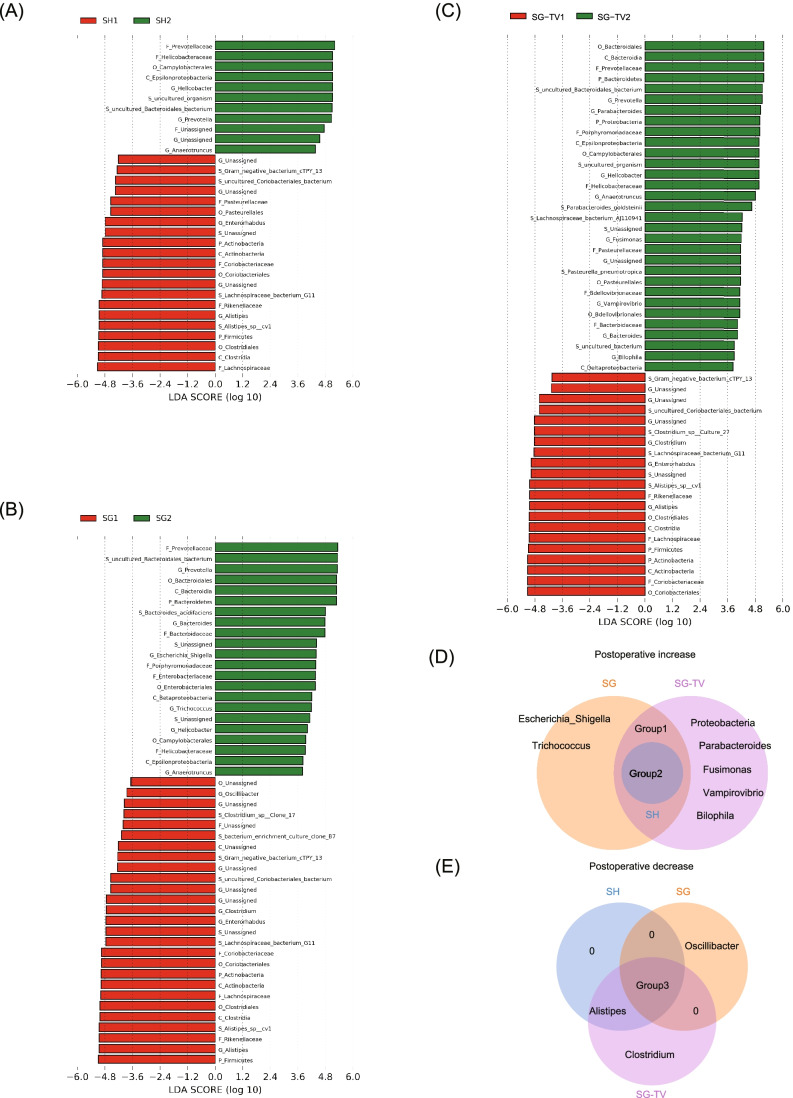
The microbiota profile differs between pre- and post-surgery samples in SH (A), SG (B), and SG-TV (C) group. Significance obtained by LDA score > 2. The Venn diagram compares and contrasts the number of taxa in phylum and genus levels that increased (D) and decreased (E) significantly after treatments with SH, SG, and SG-TV (all LDA score > 2). 1, pre-surgery; 2, post-surgery. Group 1: *Bacteroidetes*, *Bacteroides*; Group 2: *Prevotella*, *Helicobacter*, *Anaerotruncus*; Group 3: *Firmicutes*, *Actinobacteria*, *Enterorhabdus*

In order to further investigate the impact of different surgeries on gut microbiota, LEfSe analysis was conducted to identify discriminative taxa. The taxas 32, 49, and 52 were identified in SH, SG, and SG-TV groups, respectively (Fig. 5A–C). Notable, there were several unique genera or phyla in SG and SG-TV groups (Fig. 5D, E). We found that genera *Escherichia_Shigella* and *Trichococcus* increased, and *Oscillibacter* decreased specifically in the SG group. Besides, phylum *Proteobacteria* and genera *Parabacteroides*, *Fusimonas*, *Vampirovibrio* and *Bilophila* increased, while genus *Clostridium* decreased specifically in the SG-TV group.

Among these different microbes, genera *Escherichia_Shigella*, *Parabacteroides*, and Vampirovibrio also showed negatively correlated with BW and FBG (Fig. 4), which suggested these microbes may be the potential contributors to stable weight loss and FBG reduced after bariatric surgery.

## Results Compared with the Prior

To further confirm that these different microbes were indeed affected by surgery, we made a longitudinal comparison with related results based on sufficient literature research (Table 1). The results showed that changes of phyla *Proteobacteria*, *Firmicutes* and *Bacteroidetes* and genus *Bacteroides* varies in different studies, six genera including *Vampirovibrio*, *Trichococcus*, *Helicobacter*, *Fusimonas*, *Escherichia_Shigella*, *Enterorhabdus*, and *Bilophila* have not been confirmed in the prior studies, and change of genus *Alistipes* was inconsistent with previous studies. It is worth noting that changes of phylum *Actinobacteria*, genera *Prevotella*, *Parabacteroides*, *Oscillibacter*, *Clostridium*, and *Anaerotruncus* after surgeries were consistent with the present study, which indicated that these genera were indeed significantly affected by surgery. But considering that *Parabacteroides* was the only genus that is not present in the control group (SH) and showed negatively correlated with BW and FBG (Fig. 4), *Parabacteroides* may play important roles in post-operative weight loss.

**Table 1:** Longitudinal comparison of gut microbiome affected by different bariatric surgeries

Taxa	The present	The prior	Ref
P_Proteobacteria	SH-TV↑	RYGB↑, SG↑↓, LGB↑	8,13,5
P_Firmicutes	SH↓, SG↓, SG-TV↓	SH↑, RYGB↓, SG↓	11,12,5
P_Bacteroidetes	SG↑, SG-TV↑	RYGB↑↓	12,9,5
P_Actinobacteria	SH↓, SG↓, SG-TV↓	RYGB↓,	12
G_Vampirovibrio	SG-TV↑		
G_Trichococcus	SG↑		
G_Prevotella	SH↑, SG↑, SG-TV↑	RYGB↑, SG↑	15
G_Parabacteroides	SG-TV↑	RYGB↑, SG↑	14
G_Oscillibacter	SG↓	BS↓	6
G_Helicobacter	SH↑, SG↑, SG-TV↑		
G_Fusimonas	SG-TV↑		
G_Escherichia_Shigella	SG↑		
G_Enterorhabdus	SH↓, SG↓, SG-TV↓		
G_Clostridium	SG-TV↓	BS↓	6
G_Bilophila	SG-TV↑		
G_Bacteroides	SG↑, SG-TV↑	RYGB↑↓,	11,15
G_Anaerotruncus	SH↑, SG↑, SG-TV↑	RYGB↑, SG↑	15
G_Alistipes	SH↓, SG-TV↓	RYGB↑, SG↑	14,15

*SH* sham surgery, *SG* sleeve gastrectomy, *TV* truncal vagotomy, *RYGB* Roux-en-Y gastric bypass, *BS* bariatric surgery. ↑, significantly increased; ↓, significantly decreased

## Functional Analysis of Microbes after Different BS

**Fig. 6:**
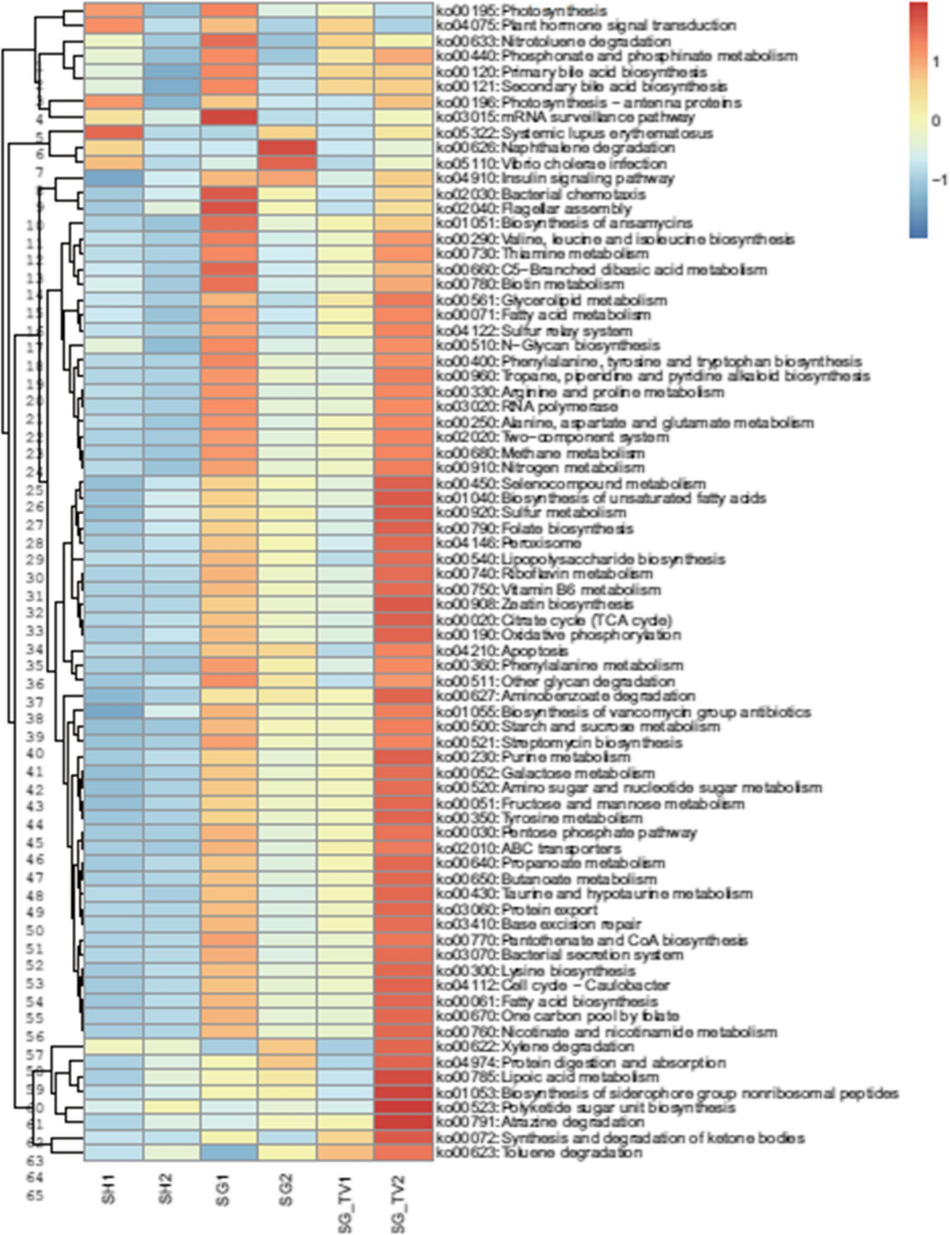
Differences in KEGG pathway after different bariatric surgeries. 1, pre-surgery; 2, post-surgery

Furthermore, we explored the effects of bariatric surgery on metabolism of gut microbiota. The KEGG pathways involved in each sample were predicted using the pircust2 software. A total of 76 pathways were identified to be significantly different before and after surgery process (Fig. 6). Heatmap showed that almost all KEGG pathways showed higher abundances after surgery in the SG-TV group, indicating an increasing activity of gut microbiota, and this result corroborated the above findings of the proliferation of gut microbiota after surgery. In addition, we found the abundances of polyketide sugar unit biosynthesis were higher in the SG-TV group, and Pearson correlation analysis also showed significant negatively correlated with BW (*p* = 0.025) and FBG (*p* = 0.016) (Fig. S1).

## Discussion

There are increasing evidences for the role of gut microbes in post-operative weight loss [4]. In this study, we have explored the impacts of three BSs on weight loss and FBG level, as well as the short-term changes in gut microbiota composition and metabolic pathways after different BSs. It is worth noting that TV-related surgeries (SG-TV) were the first time to be studied. The beneficial effects of SG-TV were reinforced, and the changes in gut microbiota following SG-TV were studied firstly.

First, in addition to the FBG, F/B ratio was considered as an eventual biomarker of obesity [29, 30]. However, the validity of this potential marker is disputed. Some studies have reported that the composition of gut microbiota of obese subjects present a higher *Firmicutes*/*Bacteroidetes* ratio (F/B) [31–33]. Whereas, with the rapid accumulation of data, several studies did not find any differences of this parameter between obese and normal-weight individuals or even reported decreased F/B ratio in obese subjects [34, 35]. Furthermore, several meta-analyses have clarified that the heterogeneity of the results could be due to the insufficient number of subjects including in most of the studies [36, 37]. In our study, a trend was observed in decreasing F/B ratio after SG and SG-TV relative to pre-operation, while the opposite trend was found in mice which underwent SH. The inconsistency of results may be caused by different surgical methods. Murphy et al. examined gut microbiota changes after laparoscopic RYGB and SG in obese patients and found that RYGB resulted in increased F/B ratio, while SG resulted in the opposite result [12]. This may suggest that different surgical interventions are one of the main reasons for the heterogeneity of the results. Therefore, the ratio of F/B is not a robust marker of microbiome dysbiosis associated with obesity [38].

Second, little is known about the impact of TV-related surgeries on gut microbiota. In this study, results showed that the absolute cell number obviously increased after SG-TV, and the most discriminative taxa were identified in the SG-TV group. Notably, *Parabacteroides* was identified as the SG-TV specific genus, which deserves more attention. Previous studies have already suggested the potential role of *Parabacteroides* in obesity resistance [39, 40]. It was reported that *Parabacteroides* was negatively associated with multiple significant clinical indicators about bodyweight and serum lipids [39, 41]. Furthermore, *Parabacteroides goldsteinii* and *Parabacteroides distasonis*, belonging to the to the genus *Parabacteroides* have been considered as anti-obesity bacteria [42–44]. Previous studies have reported that *P. goldsteinii* and *P. distasonis* could play predominant role in the anti-obesity effects of related medicines, and are novel probiotic bacteria that may be used to treat obesity and associated metabolic disorders [43–45]. In addition, based on the predicted PICRUSt of gut microbiota, the present study revealed many pathways related to metabolisms. Among them, polyketide sugar unit biosynthesis shows higher abundance in the SG-TV group, and significantly negatively correlated with BW and FBG (Fig. 6; Fig. S1), which was also observed in previous studies [46, 47]. Fan et al. found that *Parabacteroides* and polyketide sugar unit biosynthesis were significantly increased at the same time, which was consistent with our results [48]. However, the potential correlation between them remains to be explored. In a word, the genus *Parabacteroides* may be a potential contributor to the beneficial effects of TV-related surgery, and the potential metabolic function need to be further verified.

We acknowledge several limitations of this study. Firstly, the sample size was limited but is comparable to other animal bariatric gut microbiota studies reported to date using 16S rRNA sequencing [10, 11]. Secondly, we use predicted rather than sequenced bacterial functions. Thus, metabolomics data are needed to explore these preliminary findings in further detail. Thirdly, the frequency of sampling collection is low; a long-term sampling was considered in our follow-up researches.

In conclusion, SG-TV could achieve a better effect of weight loss, indicating the synergistic effect of SG combined with TV. Besides, significant pre- to post-surgeries changes in the gut microbiome diversity, composition, and metabolic pathways were observed. And we found that the changes of the gut microbiome in a procedure-related manner. Moreover, the genus *Parabacteroides*, which is enriched specifically after SG-TV and negatively correlates with post-operative BW and FBG, may be the potential contributors to weight loss.

## Supplementary Information

Fig. S1 Correlation between postoperative weight or blood glucose and KEGG pathway. Only the KEGG pathway with significant differences were showed (p < 0.05) (PNG 82 kb)

High Resolution Image (EPS 1430 kb)
