# Supplementary figures and images for: The Genus Parabacteroides Is a Potential Contributor to the Beneficial Effects of Truncal Vagotomy–Related Bariatric Surgery
Source: Obes Surg. 2022 May 11;32(7):1–11. doi: 10.1007/s11695-022-06017-9 (PMC9276728; doi:10.1007/s11695-022-06017-9)

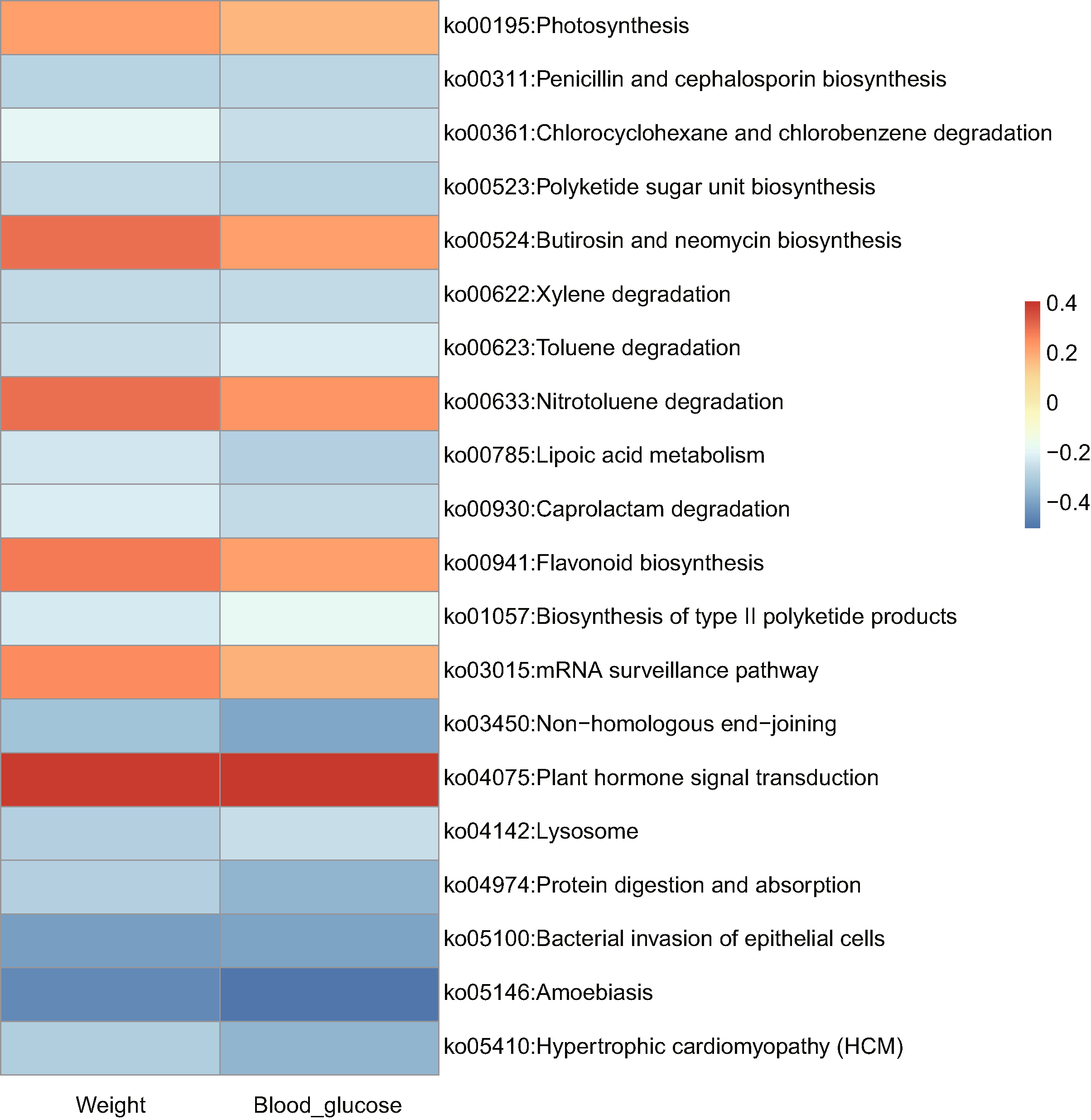

Supplement: Supplementary file 1 — Correlation between postoperative weight or blood glucose and KEGG pathway. Only the KEGG pathway with significant differences were showed (p < 0.05) (PNG 82 kb) [file 11695_2022_6017_Fig7_ESM.png]
